# Effect of Dietary Supplementation of Organic Acids on Performance, Intestinal Histomorphology, and Serum Biochemistry of Broiler Chicken

**DOI:** 10.4061/2010/479485

**Published:** 2010-06-14

**Authors:** Sheikh Adil, Tufail Banday, Gulam Ahmad Bhat, Masood Saleem Mir, Manzoor Rehman

**Affiliations:** ^1^Department of Livestock Production and Management, Faculty of Veterinary Sciences and Animal Husbandry, Sher-e-Kashmir University of Agricultural Sciences and Technology Kashmir, Alusteng 190006, India; ^2^Department of Veterinary Pathology, Faculty of Veterinary Sciences and Animal Husbandry, Sher-e-Kashmir University of Agricultural Sciences and Technology, Kashmir, Alusteng 190006, India; ^3^Department of Veterinary Biochemistry, Faculty of Veterinary Sciences and Animal Husbandry, Sher-e-Kashmir University of Agricultural Sciences and Technology, Kashmir, Alusteng 190006, India

## Abstract

The aim of the study was to determine the effect of dietary supplementation of organic acids on the performance, intestinal histomorphology, and blood biochemistry of broiler chicken. The birds in the control (T_1_) group were fed the basal diet whereas in other treatment groups basal diet was supplemented with 2% butyric acid (T_2_), 3% butyric acid (T_4_), 2% fumaric acid (T_4_), 3% fumaric acid (T_5_), 2% lactic acid (T_6_), and 3% lactic acid (T_7_). Broiler chicken fed diets supplemented with organic acids had significantly (*P* < .05) improved body weight gains and feed conversion ratio. No effect (*P* < .05) on cumulative feed consumption was observed. The addition of organic increased villus height in the small intestines but the differences were not significant (*P* < .05) in case of the ileum. Serum calcium and phosphorus concentrations were increased (*P* < .05) but no effect (*P* < .05) on the concentration of serum glucose and cholesterol, serum glutamic pyruvic transaminase (SGPT), and serum glutamic oxaloacetate transaminase (SGOT) was observed. The results indicated that the organic acid supplementation, irrespective of type and level of acid used, had a beneficial effect on the performance of broiler chicken.

## 1. Introduction

A modernistic challenge in the poultry production is to exploit the use of specific dietary supplements to boost the intrinsic potential of poultry bird to perform better. Following the ban on the use of antibiotics as growth promoters in animal nutrition by the European Union (EU) in 2006, the nutritionists and researchers attempted other alternatives claiming to enhance the performance of broiler chicken. One such alternative was the use of organic acids as feed additives in the animalproduction.

 Organic acids and their salts are generally regarded as safe (GRAS) and have been approved by most member states of EU to be used as the feed additives in animal production. The use of organic acids has been reported to protect the young chicks by competitive exclusion [1], enhancement of nutrient utilization and growth and feed conversion efficiency [2]. The organic acids in nondissociated (nonionised, more lipophilic) form can penetrate the bacteria cell wall and disrupt the normal physiology of certain types of bacteria [3]. Apart from the antimicrobial activity, they reduce the pH of digesta, increase the pancreatic secretion, and have trophic effects on the mucosa of gastro-intestinal tract [4]. Organic acids have made a great contribution to the profitability in the poultry production and also provided people with the healthy and nutritious poultry products [5–7]. Acidification with various organic acids has been reported to reduce the production of toxic components by the bacteria and colonization of pathogens on the intestinal wall, thus preventing the damage to epithelial cells [8], also improve the digestibility of proteins, calcium, phosphorus, magnesium, and zinc, and serve as substrates in the intermediary metabolism [9].

 The present study was conducted with the objectives to evaluate the effect of dietary supplementation of organic acids on the performance, intestinal histomorphology, and serum biochemistry of the broiler chicken.

## 2. Materials and Methods

The study was carried out utilizing 315 Cobb straight run commercial broiler chicks. The experimental protocol was approved by Institutional Animal Ethics Committee vide no. AU/DRI/PF/3161-62. On arrival, the chicks were provided with 8% sugar solution and ground maize for the first 12 hours. To avoid stress, the water soluble vitamins and electrolytes were added to the drinking water for the first 3 days. At 7 days of age, the birds were individually weighed and randomly assigned into the seven groups having three replicates of 15 chicks each. The birds were placed in the battery cages, and the temperature was controlled and gradually reduced from 32°C to 20°C on day 42. The chicks were maintained on a 24-hour consistent lighting schedule. Proper ventilation was ensured by means of the exhaust fans. The birds were vaccinated against New castle and Gumboro's diseases. A fresh feed and water were provided daily ad libitum. The feeding programme consisted of a starter diet until 21 days and a grower diet until 42 days of age. The birds in the control group were given a diet without additives (T_1_). The ingredient and chemical composition of the control diet are listed in Table 1. The chemical analysis was done as per the AOAC, 1996. The other six treatment groups were given the same diet as fed to the control group but was supplemented with 2% butyric acid (T_2_), 3% butyric acid (T_4_), 2% fumaric acid (T_4_), 3% fumaric acid (T_5_), 2% lactic acid (T_6_), and 3% lactic acid (T_7_). The feed ingredients were always properly mixed and prepared in lots of 60 kgs for each treatment. The organic acids in powder form were mixed thoroughly in aforesaid quantities to a small amount of feed (1 kg) in a premixer. The resultant mixture was then mixed with the rest of the feed in a mechanical blender until a thorough and consistent mixture was obtained.

The body weight of birds per replicate was recorded on the individual basis at weekly intervals. The cumulative feed consumption per replicate was also recorded on the weekly basis. Feed conversion ratio per replicate was worked out at weekly intervals by taking into consideration the weekly body weight gain and the feed consumption of respective replicate.

At the end of the feeding trial, six birds per treatment were selected at random and utilized for the carcass evaluation study. Each bird was weighed immediately before severing the jugular vein at the atlantooccipital joint and then allowed to bleed. The shanks were cut off at the hock joint, and carcass was subjected to the scalding process at 60°C for 30 seconds. The feathers were removed completely by hand picking leaving the skin intact. Thereafter, the abdominal cavity was opened to expose the visceral organs, and the carcass characteristics were evaluated.

For the histopathological analysis, the tissue samples from the duodenum, jejunum, and the ileum were collected from the slaughtered birds and fixed in 10% buffered formalin saline. Tissues were dehydrated by immersing through a series of alcohols of increasing concentrations (from 70% to absolute), infiltrated with xylene, and embedded in paraffin. Casting of blocks was carried out in L-molds (two L-shaped pieces) which facilitated the manipulation of size as per the requirement. The rotary type microtome was used for cutting the paraffin sections. The blocks were properly trimmed and the sections of 5 mm thickness were cut. Continuous ribbons (6-7 inches long) of the material were cut and laid on the surface of constant temperature water bath (around 55°C). The sections were separated with a heated scalpel after they spread completely. The cut sections were mounted on the clean glass slides using Mayer's egg albumin as the section adhesive.The mounted slides were dried in paraffin oven at 60°C for one hour. The tissue sections were stained by the Harris haematoxylin and eosin staining method. The paraffin sections were deparaffinised with the xylene before hydration through graded alcohol to distilled water. This was followed by the dehydration in ascending grades of alcohol. The clearing was performed in the xylene and a drop of Distrene Plasticiser Xylene (DPX) mountant was placed on a cover slip and the section on the slide pressed on it. The slide was inverted and the cover slip was pressed with a rod to remove the air bubbles, if any trapped. The values were measured with an oculometer at a magnification of 10x under a light microscope fitted with the stage micrometer.

Blood samples were collected from the slaughtered birds in nonheparinised tubes. The samples were centrifuged at 3000 rpm for 15 minutes, and the serum obtained was stored at −20°C until analysis. SGPT, SGOT, serum glucose, and cholesterol were determined by the auto analyzer using commercially available kits purchased from the Accurex biomedical company. The serum calcium and phosphorus were determined calorimetrically by using the kits purchased from Crest biosystems company.

The data obtained was statistically assessed by the analysis of variance (ANOVA) through General Linear Model procedure of SPSS (10.0) software considering replicates as experimental units, and the values were expressed as means ± standard error. Duncan's multiple range test [10] was used to test the significance of difference between means by considering the differences significant at *P* ≤ .05.

## 3. Results and Discussion

The body weight gains were significantly (*P* < .05) improved by dietary supplementation of organic acids when compared with the control group (Table 2). The highest weight gains were achieved in the birds fed 3% fumaric acid, followed by the group fed diet supplemented with 3% lactic acid. The 3% inclusion levels were found better in promoting the weight gains when compared with the groups fed diets supplemented with the 2% levels. The results of the present study regarding weight gains coincide with the other workers [11–14] who reported that the supplementation of organic acids in broiler chicken improved the body weight gain when compared with the unsupplemented group. The improved body weight gain is probably due to the beneficial effect of organic acids on the gut flora. The organic acids may affect the integrity of microbial cell membrane or cell macromolecules or interfere with the nutrient transport and energy metabolism causing the bactericidal effect [6]. Use of organic acid mixture decreases the total bacterial and gram negative bacterial counts significantly in the broiler chicken [15]. Besides, the butyric acid has been reported to reduce the virulent gene expression and invasiveness in *Salmonella *Enteritidis, leading to its decreased colonization in the caeca of broiler chicken [16–18]. Furthermore, organic acids supplementation has pH reducing property, although nonsignificant, in various gastrointestinal segments of the broiler chicken [19]. The reduced pH is conducive for the growth of favourable bacteria simultaneously hampering the growth of pathogenic bacteria which grow at a relatively higher pH. However, it is worth mentioning that the effects of organic acids down the digestive tract diminish because of the reduction in concentration of acids as a result of absorption and metabolism [20]. Thus, it can be hypothesized that the effect of organic acids in the distal segments of gastro-intestinal tract could be due to the reduced entry of pathogenic bacteria from the upper parts of gastro-intestinal tract as a compensatory mechanism but no valid literature regarding such mechanism was found. The beneficial microbiological and pH-decreasing abilities of organic acids might have had resulted in the inhibition of intestinal bacteria leading to the reduced metabolic needs, thereby increasing the availability of nutrients to the host. This also had decreased the level of toxic bacterial metabolites as a result of lessened bacterial fermentation, causing an improvement in the protein and energy digestibility, thus ameliorating the weight gain and performance of experimental birds. Moreover, the organic acids improve the villus height in the small intestines (Table 3) and also have a direct stimulatory effect on the gastro-intestinal cell proliferation as reported by Tappenden and McBurney [21] that short chain fatty acids increase plasma glucagon-like peptide-2 (GLP-2) and ileal proglucagon mRNA, glucose transporter (GLUT2) expression, and protein expression, which are all signals which can potentially mediate gut epithelial cell proliferation. These histological changes in small intestines probably had increased the intestinal surface area, facilitating the nutrient absorption to a greater extent and, thus boosted the growth promoting effect of organic acid supplementation.

 The feed consumption was found statistically non-significant (*P* > .05) among all the treatment groups (Table 2). These results are in agreement with Hernandez et al. [22] who found no difference in the cumulative feed consumption between the groups fed organic acids and the control group. Chicks fed the diets supplemented with organic acids showed a significant (*P* < .05) improvement in the FCR as against the chicks fed the control diet (Table 2). The improvement in the FCR could be possibly due to better utilization of nutrients resulting in increased body weight gain (Table 2) in the birds fed organic acids in the diet. These results are in concordance with the reports of earlier researchers [23, 24] who reported that the supplementation of organic acids improved the feed conversion ratio in broiler chicken. The carcass characteristics of broiler chicken fed diets supplemented with the organic acids showed no significant differences (*P* > .05) between various treatment groups (Table 2), confirming the earlier findings [25].

**Figure 1:**
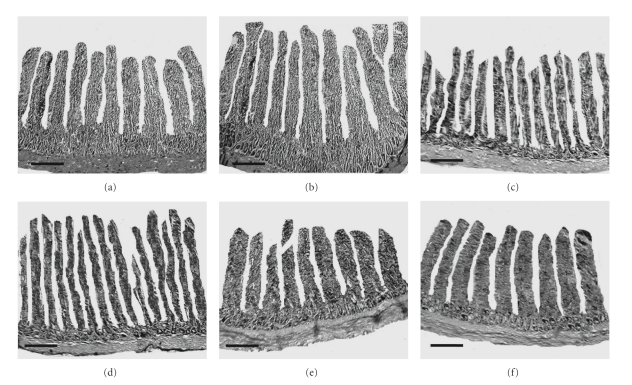
The sections of the duodenum, jejunum, and ileum showing villus height in control group and the birds supplemented with different organic acids. Hematoxylin and eosin staining. Scale bars represent 260 *μ*m.Villus height in the duodenum of broiler chicken in control group (a) and 3% butyric acid group (b). Villus height in the jejunum of broiler chicken in control group (c) and 3% fumaric acid group (d). Villus height in the ileum of broiler chicken in control group (e) and 2% fumaric acid group (f).

 The mean values regarding the histomorphological alterations in the broiler chicken fed organic acid based diets are given in Table 3. Dietary supplementation of organic acids significantly (*P* < .05) increased the villus height in the duodenum, jejunum, and ileum but the values were significant (*P* < .05) only in the duodenum and jejunum when compared with the control group. The highest duodenal, jejuna, and ileal villus heights were recorded in the birds fed diets supplemented with 3% butyric acid, 3% fumaric acid, and 2% fumaric acid, respectively (Figure 1). These results are in harmony with the earlier workers [26, 27] who reported increased villus heights in duodenum and jejunum with most of the organic acidifiers which they attributed to the fact that organic acids reduce the growth of many pathogenic or nonpathogenic intestinal bacteria, decreasing the intestinal colonization and infectious processes, ultimately decreasing the inflammatory reactions at the intestinal mucosa, which increases the villus height and functions of secretion, digestion, and absorption of nutrients by the mucosa. The crypt depth in the duodenum, jejunum, and ileum was not affected (*P* > .05) among different treatment groups. Moreover, the muscularis thickness was decreased in all the segments of small intestines but the differences were not significant (*P* > .05) when compared with the control group. The reduction in the muscularis thickness is helpful in improving the digestion and absorption of nutrients as reported by Teirlynck et al. [28] that the thickening of mucous layer on the intestinal mucosa contributes to the reduced digestive efficiency and nutrient absorption. 

 The mean values of serum constituents in broiler chicken fed organic acid supplemented diets are shown in Table 4. Supplementation of organic acids showed no significant (*P* > .05) difference in the concentration of serum glucose and cholesterol among all the treatment groups including the control group confirming the earlier findings [22] that organic acid supplementation had no effect on the blood metabolites in the broiler chicken. Results of serum calcium and phosphorus showed that the chicks fed diets supplemented with organic acids had higher (*P* < .05) concentrations when compared with the chicks fed control diet which could be attributed to the fact that acidic anion complexes with the minerals like calcium and phosphorus result in an improvement in the digestibility of these minerals as reported by several workers [29–31]. Snow et al. [32] reported that addition of citric acid to broiler diets improved the tibia ash without reducing the weight gain or feed intake. There was no significant (*P* < .05) difference in the SGPT and SGOT levels between the chicks fed diets supplemented with organic acids and the control group as observed by Abdel Fattah et al. [19] who concluded that dietary supplementation of organic acids could be done up to the level of 3% in the diet of broiler chicken without causing any adverse effect on the kidney and liver functions.

 In conclusion, dietary organic acids may be exploited as growth promoters in the broiler chicken as in the present study they had positive outcome on the performance, irrespective of the type and level of acid used, possibly because of their beneficial antimicrobial effect apart from positive impact on histology of the small intestines, thereby facilitating the nutrient absorption and growth performance in broiler chicken.

## Figures and Tables

**Table 1:** Ingredient and chemical composition of experimental basal diets.

Ingredients (%)	Starter (up to 3 wks)	Grower (3–6 wks)
Maize	52.60	59.70
Soya bean meal	35.80	32.10
Fish meal	8.50	5.10
Limestone	1.28	1.50
DCP	0.84	0.85
vitamin premix*	0.19	0.19
Trace mineral mixture**	0.23	0.23
Salt	0.30	0.30
L-lysine	0.08	0.03
DL-methionine	0.18	0.10

Analyzed Values:		
Crude protein %	22.49	20.22
Crude fiber %	4.915	4.997
Ether extract %	7.235	8.614
Total ash %	4.013	3.731

Calculated Values:		
Metabolizable energy (Kcal/Kg diet)	2861.12	2933.92
Calcium %	1.486	1.294
Available phosphorus %	0.756	0.687
Lysine %	1.292	1.073
Methionine %	0.581	0.460

*Vitamin premix (per 2.5 kg of diet): vitamin A 15.000 IU, vitamin D3 1.500 IU, vitamin E 20 mg, vitamin K3 5 mg, vitamin B1 3 mg, vitamin B2 6 mg, niacin 25 mg, vitamin B6 5 mg, vitamin B12 0.03 mg, folic acid 1 mg, D-biotin 0.05 mg, Ca-D- pantothenate 12 mg, carophyll-yellow 25 mg, and choline chloride 400 mg.

**Trace mineral premix (per kg of diet): Mn 80 mg, Fe 60 mg, Zn 60 mg, Cu 5 mg, Co 0.2 mg, I 1 mg, and Se 0.15 mg.

**Table 2:** Effect of organic acid supplementation on the performance and carcass characteristics of broiler chicken.

Parameters	Treatment groups							Sig.
	T_1_	T_2_	T_3_	T_4_	T_5_	T_6_	T_7_	
Final body weight gain (g)	1525.4^a^ ± 23.6	1629.8^bc^ ± 27.6	1666.7^bc^ ± 22.1	1631.8^bc^ ± 29.9	1704.2^b^ ± 26.3	1602.4^c^ ± 32.6	1673.0^bc^ ± 26.8	∗
Feed consumption (g)	3081.3 ± 53.0	2998.6 ± 56.3	3083.3 ± 43.3	2986 ± 41.9	3118.6 ± 63.7	2964.3 ± 113.8	3078.3 ± 40.9	NS
Feed conversion ratio	2.02^a^ ± 0.03	1.84^b^ ± 0.03	1.85^b^ ± 0.05	1.83^b^ ± 0.05	1.83^b^ ± 0.02	1.85^b^ ± 0.11	1.84^b^ ± 0.03	∗
Dressing percentage	70.79 ± 0.63	71.74 ± 0.57	72.70 ± 0.79	71.77 ± 1.35	72.07 ± 0.97	70.77 ± 1.43	71.30 ± 0.38	NS
Gizzard weight (g)	38.33 ± 0.88	36.33 ± 2.72	38.0 ± 3.05	43.33 ± 2.33	40.66 ± 0.33	42.66 ± 4.09	41.66 ± 4.40	NS
Heart weight (g)	10.33 ± 0.33	10.0 ± 0.57	12.0 ± 1.52	10.2 ± 1.00	11.0 ± 1.15	11.6 ± 1.52	10.55 ± 0.66	NS
Liver weight (g)	41.66 ± 3.17	49.0 ± 2.51	47.33 ± 0.88	50.0 ± 1.73	44.66 ± 1.20	51.66 ± 6.00	41.33 ± 4.05	NS
Blood weight (g)	127.3 ± 16.3	126.0 ± 23.4	114.3 ± 16.8	123.3 ± 16.0	137.6 ± 31.7	146.0 ± 16.2	123.0 ± 3.60	NS
Feather weight (g)	190.6 ± 24.9	212.6 ± 17.1	184.3 ± 17.3	186.6 ± 9.82	172.6 ± 31.4	201.0 ± 19.0	198.0 ± 5.29	NS
Head weight (g)	50.0 ± 5.77	53.3 ± 3.33	62.0 ± 8.66	58.3 ± 4.40	55.0 ± 7.63	56.6 ± 12.0	50.0 ± 5.57	NS

*Means within the same row with different superscripts are significantly different (*P* ≤ .05). NS: Non significant.

**Table 3:** Effect of organic acid supplementation on histomorphology of small intestines of broiler chicken.

Parameter	Treatment Groups							Sig.
	T_1_	T_2_	T_3_	T_4_	T_5_	T_6_	T_7_	
*Duodenum * ** (** *μ*m)								
Villus height	1166.88^a^ ± 56.32	1252.51^ac^ ± 29.83	1410.38^bc^ ± 46.41	1237.84^ac^ ± 24.85	1378.05^bc^ ± 20.55	1161.40^a^ ± 30.85	1321.61^c^ ± 38.53	∗
Crypt depth	175.48 ± 29.95	178.20 ± 3.12	169.16 ± 11.11	165.71 ± 7.17	174.81 ± 22.45	180.76 ± 1.93	179.23 ± 25.61	NS
Muscularis thickness	186.21 ± 13.71	169.66 ± 9.49	158.65 ± 10.87	172.11 ± 9.21	153.14 ± 12.11	179.69 ± 8.88	181.71 ± 16.88	NS

*Jejunum * ** (** *μ*m)								
Villus height	984.05^a^ ± 25.77	1117.28^b^ ± 27.41	1124.72^c^ ± 28.40	1106.75^b^ ± 31.01	1256.94^c^ ± 48.08	1074.2^b^ ± 13.03	1212.95^c^ ± 21.88	∗
Crypt depth	157.83 ± 27.18	157.20 ± 24.03	163.04 ± 7.90	151.73 ± 23.25	150.31 ± 13.00	166.78 ± 15.16	144.05 ± 13.76	NS
Muscularis thickness	163.45 ± 19.65	153.81 ± 7.14	145.31 ± 7.31	151.52 ± 18.58	150.32 ± 9.35	146.27 ± 13.11	139.66 ± 12.90	NS

*Ileum * **(** *μ*m)								
Villus height	676.13 ± 49.03	739.17 ± 46.15	876.32 ± 22.37	898.85 ± 103.8	841.0 ± 29.46	749.19 ± 46.68	863.71 ± 66.42	NS
Crypt depth	152.16 ± 17.96	159.84 ± 10.95	148.27 ± 4.50	147.19 ± 5.07	155.81 ± 13.04	152.87 ± 10.81	151.65 ± 6.41	NS
Muscularis thickness	156.40 ± 15.38	132.95 ± 22.10	122.79 ± 7.54	146.85 ± 16.19	130.95 ± 21.23	149.52 ± 22.44	140.90 ± 18.73	NS

*Means within the same row with different superscripts are significantly different (*P* ≤ .05), NS: Non significant.

**Table 4:** Effect of organic acid supplementation on serum constituents of broiler chicken.

Parameter	Treatment Groups							Sig.
	T_1_	T_2_	T_3_	T_4_	T_5_	T_6_	T_7_	
Glucose (mg/dL)	183.4 ± 8.02	184.2 ± 9.34	190.2 ± 8.83	180.3 ± 8.39	186.2 ± 4.27	179.0 ± 15.41	178.9 ± 12.45	NS
Cholesterol (mg/dL)	128.3 ± 3.89	123.4 ± 2.35	125.3 ± 2.61	129.5 ± 0.91	127.3 ± 2.34	128.1 ± 2.78	122.5 ± 0.81	NS
Calcium (mg/dL)	10.70 ^a^± 0.16	11.94^b^ ± 0.10	12.20^b^ ± 0.25	11.76^b^ ± 0.32	11.97^b^ ± 0.30	11.78^b^ ± 0.21	12.14^b^ ± 0.38	∗
Phosphorus (mg/dL)	6.22^a^ ± 0.26	7.18^b^ ± 0.22	7.59^b^ ± 0.30	7.00^ab^ ± 0.13	7.42^b^ ± 0.34	6.93^ab^ ± 0.39	7.53^b^ ± 0.17	∗
SGPT (*μ*/L)	15.63 ± 0.47	14.95 ± 0.86	17.31 ± 2.04	16.623 ± 1.42	16.41 ± 0.84	15.24 ± 1.79	17.57 ± 0.65	NS
SGOT (*μ*/L)	97.79 ± 2.90	100.48 ± 5.12	97.60 ± 7.99	92.25 ± 10.04	99.10 ± 8.96	99.96 ± 4.95	95.61 ± 1.20	NS

*Means within the same row with different superscripts are significantly different (*P* ≤ .05), NS: Non significant.
